# Subcritical Water Technology for Extraction of Phenolic Compounds from *Chlorella sp.* Microalgae and Assessment on Its Antioxidant Activity

**DOI:** 10.3390/molecules22071105

**Published:** 2017-07-03

**Authors:** Siti Maisurah Zakaria, Siti Mazlina Mustapa Kamal, Mohd Razif Harun, Rozita Omar, Shamsul Izhar Siajam

**Affiliations:** 1 Department of Process and Food Engineering, Faculty of Engineering, Universiti Putra Malaysia, 43400 Serdang, Selangor; Malaysia; maisurahzakaria88@gmail.com; 2 Department of Chemical and Environmental Engineering, Faculty of Engineering, Universiti Putra Malaysia, 43400 Serdang, Selangor, Malaysia; mh_razif@upm.edu.my (M.R.H.); rozitaom@upm.edu.my (R.O.); shamizhar@upm.edu.my (S.I.S.)

**Keywords:** microalgae, subcritical water, extraction, antioxidant, phenolic

## Abstract

*Chlorella sp*. microalgae is a potential source of antioxidants and natural bioactive compounds used in the food and pharmaceutical industries. In this study, a subcritical water (SW) technology was applied to determine the phenolic content and antioxidant activity of *Chlorella sp*. This study focused on maximizing the recovery of *Chlorella sp.* phenolic content and antioxidant activity measured by 2,2-diphenyl-1-picrylhydrazyl (DPPH) assay as a function of extraction temperature (100–250 °C), time (5–20 min) and microalgae concentration (5–20 wt. %) using response surface methodology. The optimal operating conditions for the extraction process were found to be 5 min at 163 °C with 20 wt. % microalgae concentration, which resulted in products with 58.73 mg gallic acid equivalent (GAE)/g phenolic content and 68.5% inhibition of the DPPH radical. Under optimized conditions, the experimental values were in close agreement with values predicted by the model. The phenolic content was highly correlated (R² = 0.935) with the antioxidant capacity. Results indicated that extraction by SW technology was effective and that *Chlorella sp*. could be a useful source of natural antioxidants.

## 1. Introduction

Nearly all microalgae including *Chlorella sp*., *Spirulina platensis*, *Nannochloropsis* sp., and *Haematococcus pluvialis* generate various secondary metabolites (bioactive compounds) to aid their growth in extreme environments. Bioactive compounds are valuable, particularly for their health benefits and nutraceutical effects, such as antioxidant, antiallergic, anticancer, and anticoagulant activities. Microalgae are natural sources of biologically active compounds, such as phycobilins, fatty acids, vitamins, and sterols. They have been described as secreting a wide range of compounds with the potential to be employed as functional ingredients, including phenols, carotenoids, and other antioxidant pigments [1].

*Chlorella sp.* has been used and commercialized due to its nutrient content and other advantages, particularly its beneficial health effects, such as antioxidant, antiviral, and antitumor activities. *Chlorella sp.* is a type of microalgae that is normally found in freshwater environments. It is a unicellular photosynthetic microalgae containing green photosynthetic pigments chlorophyll in its chloroplast, and also lutein and other primary carotenoids, such as α-carotene and β-carotene [2]. Exploration of this kind of microalgae can also provide vitamins, biofuels, proteins, and polyphenols. The utility of compounds in *Chlorella sp*. has attracted continuous research into their functional properties for biological and food applications. It was confirmed that these microalgae products also have considerable amounts of phenolics, which are comparable to the phenolic content in other plant sources. Phenolic compounds have the ability to donate a hydrogen atom or electron to form stable radical intermediates, and they are major contributors to antioxidant capacities. Therefore, *Chlorella sp.* microalgae may have important and broad applications in the pharmaceutical and food industries because of the high antioxidant activities of their extracellular substances [3].

Recently, subcritical water (SW) extraction has become an increasingly popular alternative technology for the extraction of natural bioactive compounds from natural sources [4]. This technology uses subcritical water, also referred to as superheated water, pressurized hot water, or hot liquid water, as the extraction solvent. Water is inexpensive and environmentally friendly, and is therefore an ideal solvent for industrial extractions. SW extraction applies high pressure to keep water liquid beyond its normal boiling point, at temperatures between 100 and 374 ?C, and pressures below the critical pressure of 22 MPa, during extraction. Within a specific temperature and pressure range, the polarity, viscosity, surface tension, and dielectric constant of SW are significantly lower compared with water under ambient conditions. Compared with conventional extraction methods, such as hydro-distillation and organic solvent extraction, SW has many advantages, including lower extraction time, simplicity, lower operational cost, higher extract quality, and excellent environmental credentials [4].

SW has been applied for the extraction of high phenolic contents from various materials, including plants and algae. Phenolic compounds comprise a major class of plant secondary metabolites that are broadly distributed and have abundant structural diversity [5]. These compounds occur as glycones, glycosides, monomers, free or matrix-bound compounds, or well-polymerized structures [5,6]. Furthermore, they are not uniformly distributed in plant/algae matrices and their stabilities vary significantly. These factors complicate their extraction and isolation processes. Therefore, optimization of extraction procedures is required, depending on the nature of the sample and the target analytes. Some studies have reported the extraction of phenolic compounds from various plants and microalgae sources using SW, including mango leaves [7], pomegranate [8], rice bran [9], potato peel [10], cinnamon [11], citrus pomaces [12], golden oyster mushroom [13], oregano [14], marigold flower [15], and *Haematococcus pluvialis* microalga [16]. These reports indicated that SW extraction is a promising technique for the preparation and successful isolation of phenolic compounds from various matrices.

No studies have been conducted regarding response surface optimization of the extraction of phenolic compounds and antioxidant activity from *Chlorella sp.* microalgae. Therefore, this research aimed to optimize experimental conditions to obtain high phenolic content and antioxidant-rich natural extracts from *Chlorella sp.* microalgae by SW technology. The effects of extraction variables (temperature, time, and microalgae concentration) and the relationship between phenolic content and antioxidant activity from *Chlorella sp.* were also investigated.

## 2. Results and Discussion

### 2.1. Extraction Optimization

Analysis of variance (ANOVA) for the responses of *Chlorella sp.* phenolic content and antioxidant activity was used to investigate the effects of each independent variable in the SW condition parameters to construct an empirical model that maximized the phenolic content and antioxidant activity from the microalgae. This model was also used to optimize each independent variable, namely extraction temperature, time, and microalgae concentration, during SW extraction. Table 1 shows the experimental design employed, while Table 2 summarizes the phenolic content and antioxidant activity data from all extracts examined.

Analysis of variance (ANOVA) results from the response quadratic model with the highest degree of polynomial. It gave values of the model term tested for adequacy and fitness, as shown in Table 3.

Statistical analysis indicated that the proposed regression model for yield and antioxidant activity was adequate, possessing no significant lack of fit and with satisfactory values of R² (multiple correlation coefficient) for all responses. The R² values were 0.9765 and 0.9585 for both phenolic content and antioxidant activity, respectively. The closer the value of R² to unity, the better the empirical model fits actual data [17].

The significance of each term at a specified level of confidence was determined by examining its respective *p*-value and *F*-value. In fact, the *p*-value is the smallest level of significance that could be used to reject the null hypothesis, H_0_. Therefore, the smaller the value is, the more significant its corresponding coefficient and the contribution towards the response variable. From the ANOVA in Table 3, it was observed that some of the variables were highly significant to the regression model as indicated by the high *F*-value. As can be seen in Table 3, based on the *F*-values, three linear factor terms (X_1_, X_2_, X_3_), one quadratic term (X_1_^2^), and two interaction factors (X_1_X_2_, X_2_X_3_) for the phenolic response, and two linear factor terms (X_1_, X_2_), one quadratic term (X_1_^2^), and one interaction factor (X_1_X_2_) for antioxidant activity, had the largest effect on the investigated responses at a 95% confidence level, as indicated by the low *p*-value (<0.05) and the high *F*-value.

The *p*-value of each of the other terms was greater than 0.05, which indicated that the effect of these terms on the response model was not statistically significant at the 95% confidence level. In other words, only the model terms with *p*-values less than 0.05 were determined to be significant to the model equation. The insignificant model terms were removed to improve the model. Model reduction involves this type of backward elimination procedure for all the insignificant terms, to ultimately produce a new and improved experimental model. The ANOVA analysis of the reduced model (new model) equation after eliminating the insignificant terms for both phenolic content and antioxidant activity is shown in Table 4. In addition, the adequacy of the models was further justified through ANOVA. The R-squared value in the reduced model for phenolic content was 0.9679 and 0.9499 for antioxidant activity, indicating an excellent agreement between the experimental and predicted results. In addition, as shown in Table 4, the model resulted in an *F*-value of 51.61 for phenolic content and 41.11 for antioxidant activity, with an extremely low *p*-value (<0.0001), implying that the model was highly significant and was adequate for the response variables that were tested. By performing multiple regression analysis on the experimental data, the model for the response variable could be expressed using the following quadratic polynomial equation in the form of coded values, after exclusion of the insignificant terms as shown in Table 5.

#### Effect of Extraction Conditions on Phenolic Content and Antioxidant Activity

**Figure 1:**
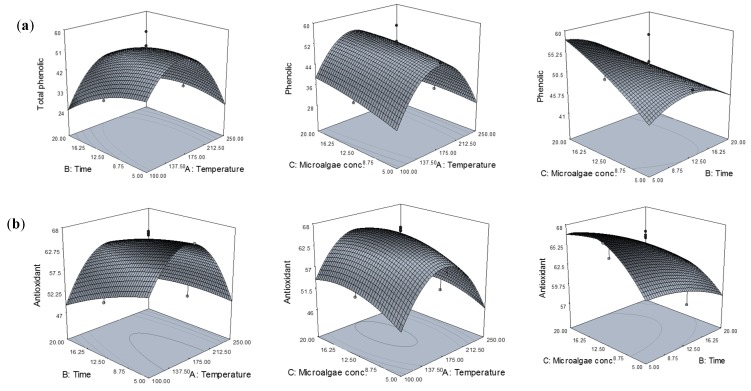
Response surface and contour plots for (**a**) phenolic content and (**b**) antioxidant activity as functions of temperature, time, and solid loading. The value of the missing independent variable in each plot was kept at the centre point.

Three-dimensional representations of the response surfaces generated by the model are shown in Figure 1. For the process with three variables, where two variables are illustrated in three-dimensional surface plots, the third variable is kept at the centre point. Varying the temperature during SW treatment allowed the solubility of different phenolic compounds to be modified. To obtain the highest phenolic content, it was important to determine appropriate operating conditions. Figure 1a shows the phenolic content as a function of extraction temperature, time, and microalgae concentration. The temperature had a large effect on the phenolic content extracted. Moreover, water at different temperatures during the SW extraction process had different dielectric constants, resulting in different polarities. Therefore, results might be related to water polarity and the solubility of phenolic compounds in *Chlorella sp.* Increasing temperature led to a gradual increase in phenolic content above 100 °C, reaching a maximum at around 175 °C. Therefore, increasing the temperature improved total phenolic yield. Indeed, a higher temperature increased the solubility and diffusion coefficients of phenolic compounds, allowing a higher extraction rate [18]. Furthermore, during the process, the viscosity and surface tension of the water were also reduced, whereas diffusivity is increased, allowing better penetration of the solvent into the matrix and enhancing the extraction process, in terms of both efficiency and speed. Therefore, mass transfer from the solid phase to the SW was improved. However, the amount of phenolic content began to decline above 175 °C due to some families of phenolic compounds becoming denatured beyond certain temperatures. In addition, previous studies have reported that, degradation of phenolic compounds was observed above 180 °C [10]. Therefore, a temperature of 175 °C was considered adequate for the extraction of phenolic compounds from *Chlorella sp.*

In terms of extraction time, it has been reported that prolonged extraction times favor the extraction of the phenolic compounds. This might be due to the time requirement for the exposure of solute or compounds to the release medium when the water penetrates into the *Chlorella sp.*, dissolves the solute, and then diffuses out from the *Chlorella sp.* However, in this study, extending the period of extraction time from 5 to 20 min led to a decrease in phenolic compound extraction. This might be due to the applied high extraction temperature causing decomposition of the phenolic compounds and structural destruction during extended extraction time. It has been reported that some families of phenolic compounds can be denatured beyond a certain temperature value [19]. In addition, due to the applied extraction high temperature, longer extraction times increase the risk of phenolic reduction by increasing the loss of phenolic by oxidation [20]. Therefore, 5 min was favored as the extraction time for phenolic compounds using SW extraction.

As shown in Figure 1a, microalgae concentration also has a significant effect on the amount of phenolic content extracted. The amount of phenolic compounds extracted increased during extraction when increasing the microalgae concentration from 5 to 20 wt. %. This might be due to an increased rate of compound mass transfer resulting from the increased microalgae concentration during the extraction. This phenomenon was attributed to the mass transfer principle. Higher solid-to-solvent ratios gave higher concentration gradients, leading to the increased diffusion and extraction yield of phenolic compounds. Therefore, a microalgae concentration of 20 wt. % was sufficient for extracting the phenolic compounds from *Chlorella sp.* by SW.

Most of the valuable characteristics of phenolic compounds are associated with their antioxidant activities [21]. The 2,2-diphenyl-1-picrylhydrazyl (DPPH) assay has been used extensively in antioxidant assays because it is fast, reliable, and reproducible, and can be used to test the general antioxidant activity of various natural substances, including algae extracts, in vitro [22]. Therefore, the DPPH radical scavenging assay has been used to monitor the capacity of extracted compounds to scavenge free radicals in hydrophilic systems. The effects of extraction temperature, time, and microalgae concentration on DPPH radical scavenging activity are shown in Figure 1b.

The effect of extraction temperature on the antioxidant activity of extracts was similar that on the phenolic content extracted. The antioxidant activity increased as the temperature increased from 100 to 175 °C. Certain antioxidant compounds might be mobilized at high temperatures, while possibly promoting concurrent decomposition of antioxidants already mobilized at lower temperatures. It has been stated that the rate of extraction of thermally stable antioxidants at elevated temperatures is higher than the rate of decomposition of less soluble antioxidants [23]. This was implied by the relatively high percent of inhibition of the extracts obtained at higher temperatures. Increasing the temperature above 175 °C during SW treatment reduced the antioxidant activity. These results showed that mobilization of the antioxidants from the substrate (algae) might occur up to a certain level, followed by their possible loss due to decomposition at higher temperatures. The antioxidant activity of the extracts was high after extraction for 5 min, but declined as the extraction time increased. As mentioned earlier, this decline was due to the longer exposure of active compounds to high temperatures that caused decomposition and structural destruction during longer extraction times. Longer extraction times increase the risk of phenolic oxidation unless reducing agents are added to the solvent system [24]. Therefore, it cause the reduction in the percent of inhibition of the extracts. Microalgae concentration during SW extraction had a similar effect on the antioxidant capacity, but with a smaller impact. The results obtained in the present study also correlated with the amount of phenolic content extracted from *Chlorella sp*., which could be responsible for the antioxidant activity observed. Based on these results, it could be concluded that the obtained *Chlorella sp.* extract with the highest phenolic content also showed the highest antioxidant activity. 

### 2.2. Optimization of Extraction Conditions

The study aimed to optimize the extraction process to maximize the extraction of phenolic compounds and antioxidant activity from *Chlorella sp*. For process optimization with two or more output responses, the concept of desirability function is useful, and was possible using the employed software. During optimization of the extraction process, some of these responses had to be maximized, while others had to be minimized, to obtain extracts of acceptable quality. In this study, both responses, phenolic compound, and antioxidant activity were maximized with 0.998 desirability. Desirability ranges from zero to one for any given response. A value of one represents the ideal case, while zero indicates that one or more responses are outside of the desirable limits. Therefore, a desirability function was developed using maximum phenolic content and antioxidant activity in the *Chlorella sp*. extracts as criteria. By applying this desirability function, optimum extraction conditions were obtained, as follows: temperature, 163 °C; time, 5 min; and microalgae concentration, 20 wt. %. This set of conditions was determined to be optimum using the response surface methodology (RSM) optimization approach and was used for experimental validation and to predict values of responses using the model equation (Table 6). Experimental values agreed with predicted values within a 95% confidence level, indicating that the model was adequate for the extraction process. 

**Figure 2:**
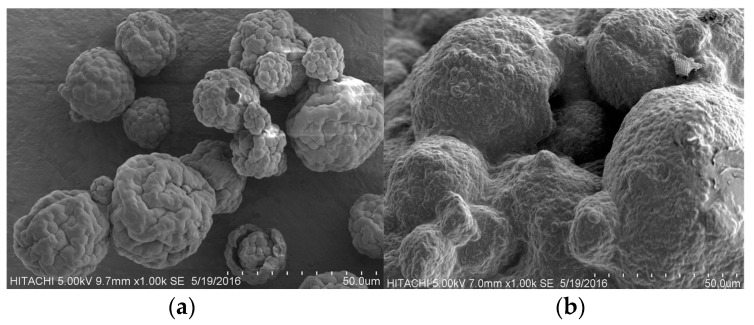
Scanning electron microscope (SEM) images of *Chlorella sp*. cell (**a**) before extraction process (untreated), and (**b**) after extraction at optimized conditions.

Figure 2 shows the surface morphology of *Chlorella sp.* cell by scanning electron microscopy (SEM) (a) before the extraction process (untreated), and (b) after extraction at optimized conditions. The untreated cells were individual rounded-shapes and agglomerated, forming a large spherical shape of the cell. Figure 2b shows the algae cells were completely ruptured and individual cells were not round in shape, compared to untreated cells of *Chlorella sp*. This shows that, SW may segregate and disrupt the microalgae cells and allow good penetration and extraction between the compounds in the algal cells, and the solvent.

### 2.3. Correlation between Phenolic Content and Antioxidant Activity of Extracts

**Figure 3:**
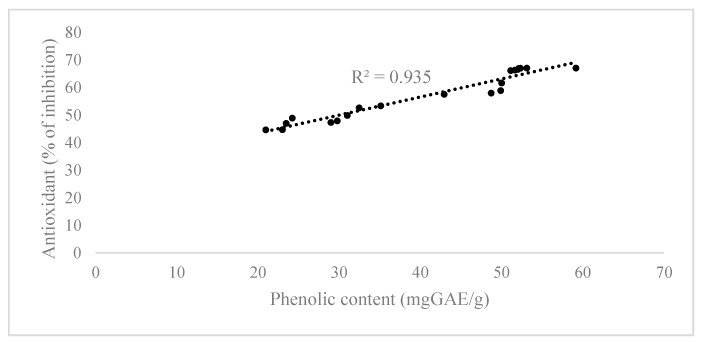
Linear regression analysis of the phenolic content with respect to antioxidant capacity towards the DPPH radical.

Using the data obtained from statistical analysis, the value of phenolic content and antioxidant activity for each operating condition during extraction was analyzed to investigate the correlation between the phenolic content extracted and the antioxidant properties of the *Chlorella sp.* extracts. The phenolic content was significantly correlated with antioxidant activity towards DPPH radicals (R² = 0.935), as shown in Figure 3. These results were in agreement with those published elsewhere [25,26]. Generally, the antioxidant capacity measured by various *in vitro* methods depends on several factors and experimental conditions, including the quantity and interactions among phenolic compounds present in the extracts, the concentration and type of free radicals, the time employed in the assay, sample dilution, pH, solubility, and stereochemical effects. Furthermore, the antioxidant activity is due to the number and acidity of phenolic hydroxyl groups and the resonance between the free electron pair on the phenolic oxygen and the benzene ring, which increases electron delocalization, conferring a nucleophilic character upon the substitution position adjacent to the hydroxyl group [27].

### 2.4. Analysis of Phenolic Acid Constituents

High-performance liquid chromatography (HPLC) analysis of the extracts obtained under optimized extraction conditions (163 °C, 5 min, 20% solid loading) was performed to confirm the results obtained by colorimetric methods and to identify the major phenolic compounds in the extracts. HPLC analysis detected and confirmed the presence of three free phenolic compounds in *Chlorella sp.* extracts, with peaks identified as corresponding to *p*-coumaric, ferulic and caffeic acids. Caffeic acid was extracted in the highest amount (2.575 mg/100 g), followed by ferulic (2.330 mg/100 g) and *p*-coumaric (2.150 mg/100 g) acids. The health effects of these phenolic acids in preventing and treating various diseases, such as flu, colds, diabetes, and cancer, have been demonstrated in several studies. Table 7 shows the different phenolic acid constituents obtained from a few plants and algae with various extraction solvents. Previous studies reported in Table 7 showed that methanol, ethanol and acetone are the most common solvents used to extract phenolic compounds from plants and algae. Through this study, it was shown that water in its subcritical condition can successfully substitute organic solvents for isolation of phenolic compounds in *Chlorella sp*. Under subcritical conditions, the intermolecular hydrogen bonds of water break down and the dielectric constant of water decreases. The dielectric constant of ethanol and of pure water at ambient temperature and pressure are 27 and 79, respectively. As the temperature increases to 250 °C, the water dielectric constant is reduced to 27, which is similar to the dielectric constant of ethanol [28]. Additionally, water, in contrast to organic solvents, is safe in terms of toxicity, flammability, and availability [29]. SW extraction (SWE) offers a series of important advantages over other techniques including high quality of extracts, a faster process, reduction of the amount of solvents and costs of the extracting agent and being an environmentally suited technique due to the use of water as the alternative to organic solvents that makes a greener extraction process [30]. This green and safe approach can be applied for use as functional food or pharmaceutical ingredients which are beneficial to health.

## 3. Materials and Methods

### 3.1. Chemicals and Materials

*Chlorella sp.* blue-green algae (derived from *Chlorella vulgaris*) was purchased from PureBulk, Roseburg, OR, USA. The dry powdered microalgae were stored in a desiccator until further use. Folin–Ciocalteu reagent, sodium carbonate, gallic acid, caffeic acid, ferulic acid, ρ-coumaric acid, acetic acid, acetonitrile, methanol, and DPPH were purchased from Sigma Aldrich, Malaysia. All chemicals were of analytical grade and used as received without further purification.

### 3.2. Experimental Design and Statistical Analysis

In this study, statistical design of experiments (DOE) was used throughout the experimental planning and data collection according to experimental matrix generated by Design Expert Version 7.0.0 (Stat Ease Inc., Minneapolis, MN, USA). This software was also used for analysis and optimization purposes. Experimental data of the phenolic content from *Chlorella sp.* were employed as Y_1_, while the antioxidant activity was employed as Y_2_, to develop an empirical model by variations of temperature (X_1_), time (X_2_), and microalgae concentration (X_3_) during SW extraction. The ranges of the independent variables and their levels are presented in Table 8.

Optimization of the SW extraction for phenolic content and antioxidant activity from *Chlorella sp.* was carried out using RSM [39]. Through this study, a three factor level of face-centered central composite design (CCD) generated a total of 20 experiments. The six replications at the design center point were utilized to provide information on the variation of response about the average and residual variance. The effects of unexplained variability in the observed response due to extraneous factors were minimized by randomizing the order of experiments. The correlation of the response to the variables studied was developed by regression model equation. Adequacy of the model developed was evaluated based on coefficients of correlation and ANOVA. ANOVA was used to show how well the model fitted the experimental data, by elucidating functional-relationship-associated statistical values. Experimental data were fitted to a second-order polynomial model and regression coefficients were obtained. The generalized second-order polynomial model used in response surface analysis was as follows:

$$Y_{k}={\;\beta }_{k0}+\overset{3}{\underset{i=1}{\sum }}\beta_{\mathrm{ki}}x_{i}+\overset{3}{\underset{i=1}{\sum }}\beta_{\mathrm{kii}}{x^{2}}_{i}+\overset{3}{\underset{i<j=2}{\sum }}\beta_{\mathrm{kij}}x_{i}x_{j}$$

where Y_k_ is the response function, β_k0_ is the center point of the system, β_ki_, β_kii_, and β_kij_ represent the coefficients of the linear, quadratic, and interactive terms, respectively, and x_i_, x_ii_, and x_i_x_j_ represent the linear, quadratic, and interactive terms of the coded independent variables, respectively.

Optimal conditions for the extraction of phenolic compounds and antioxidants from *Chlorella sp.*, depending on extraction temperature, time, and microalgae concentration, were obtained using predictive equations from RSM. The experimental and predicted values under optimized conditions were compared to determine model validity. The phenolic acid profiles of the extracts were also determined after extraction under optimized conditions. 

### 3.3 Subcritical Water Extraction

**Figure 4:**
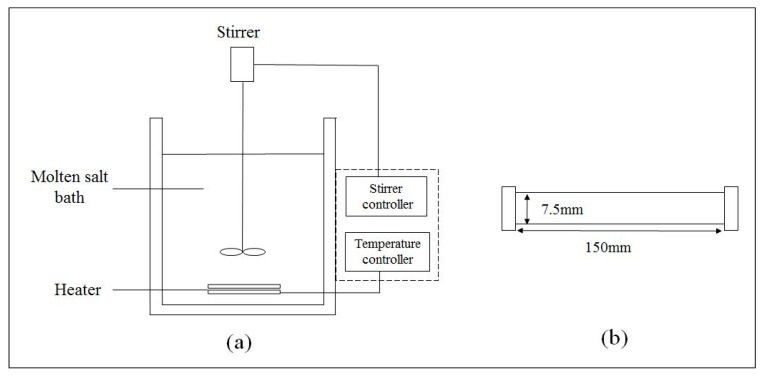
(**a**) Batch fluid extraction system and (**b**) reactor cell.

Extractions were performed using a batch fluid extraction system (Figure 4a) at extraction temperatures between 100 °C and 250 °C. The batch fluid extraction system consisted of a salt bath that was heated according to the specific temperature for the experiments. Prior to each extraction, the molten salt bath was allowed to heat up for a few minutes. Likewise, all extractions were performed in stainless steel batch reactor cells (Figure 4b) containing *Chlorella sp.* sample. The reaction cell was a stainless steel pipe (SUS316) with an inner diameter of 7.5 mm and 150 mm length.

The extraction procedure was as follows: (i) The sample was loaded into the reactor cell; (ii) the cell was filled with water solvent (5 mL); (iii) argon gas was purged into the reactor cell to release trapped air from the reactor; (iv) the reactor was immersed into a preheated molten salt bath to initiate the reaction; (v) after reaching the set extraction time, the reactor cell was removed from the bath and rapidly quenched in cooling water to terminate the reaction. The contents were then centrifuged (Hettich, Balingen, Germany) at 2376× *g* of relative centrifugal force (RCF) for 10 min and filtered through Whatman No.1 filter paper into a conical flask. The supernatant and residue were then collected for further analysis.

### 3.4. Determination of Phenolic Content

The phenolic content of the extract was determined by a modified Folin–Ciocalteu method [40]. The extract (0.2 mL) was made up to 3 mL with distilled water and mixed thoroughly with Folin–Ciocalteu reagent (0.5 mL) for 3 min, followed by the addition of 2 g/100 mL (*w*/*v*) sodium carbonate (2 mL). The mixture was allowed to incubate for a further 60 min in the dark, and absorbance was measured at 765 nm by UV-Vis spectrophotometry (Ultrospec 3100 Pro, Amersham Biosciences Corp. Piscataway, NJ, USA). The phenolic content was calculated from the gallic acid calibration curve. Gallic acid was used as a reference standard, and the results were expressed as milligram gallic acid equivalent per dry weight of microalgae (mg GAE/g). All experiments were performed in triplicate.

### 3.5. Free Radical Scavenging Capacity Using DPPH Assay

A modified DPPH free radical scavenging assay [41] was performed to determine antioxidant activity. A 0.1 mM solution of DPPH in methanol was prepared and 1 ml of this solution was added to the extracts (3 mL). After incubating in the dark for 30 min, absorbance was measured using UV-Vis spectrophotometry at 517 nm. Color changes in the mixture were observed and absorbance was then measured. The lower absorbance of the reaction mixture indicated higher free radical scavenging activity. The antioxidant activity (% of inhibition) for DPPH radicals was calculated using the following equation:

$$Antioxidant\;activity\;\left(\%\;of\;inhibition\right)=\frac{A_{0}-A_{1}}{A_{0}}\;\times \;100\%$$

where *A_0_* and *A_1_* are the absorbances of the control sample (containing all reagents except the extract sample) and the extracts, respectively. All samples were analyzed in triplicate.

### 3.6. Scanning Electron Microscopy

SEM analysis on *Chlorella sp.* cells was performed using a Hitachi S-3400N Tabletop Microscope and operated at a voltage of 5 kV. The samples were sputter-coated with gold at 5 mA for 45 s prior to SEM analysis. The images were examined under 1.00 kSE.

### 3.7. HPLC Analysis

To determine the contents of the phenolic acids, the extracts were analyzed using HPLC using Agilent G1310A pumps (Agilent Technologies, Santa Clara, CA, USA), with a diode array detector and chromatographic separations. HPLC analysis was performed using a LUNA C-18 column (5 µm, 250 mm × 4.6 mm) with a flow rate of 0.5 mL/min. The mobile phase was composed of solvent (A) water:acetic acid (94:6, *v*/*v*, pH 2.3) and solvent (B) acetonitrile. The solvent gradient was as follows: 0–15% B in 40 min, 15–45% B in 40 min, and 45–100% B in 10 min. Samples and mobile phases were filtered through a 0.22 µm Millipore filter prior to HPLC injection. Each fraction was analyzed in duplicate. The phenolic acid concentrations in the samples were identified by comparing their retention time and UV-diode array detection at 280 and 320 nm spectral data to known previously injected series of standard solutions. The values are means (*n* = 3), and they are given as mg/100 g dry weight of microalgae investigated.

## 4. Conclusions

The results in this study indicate that *Chlorella sp.* is a desirable source of phenolic compounds possessing strong antioxidant activity. Furthermore, SW technology is a suitable and environmentally friendly process that could enhance the extraction of phenolic content from this microalga. This study shows that water can be a suitable solvent to substitute organic solvents in the extraction of phenolic compounds. RSM was successfully employed to optimize phenolic extraction from *Chlorella sp*. using SW. The best extraction conditions were found to be 163 °C and 5 min with a solid loading of 20%, which afforded a phenolic content of 58.73 mg GAE/g and 68.50% inhibition of DPPH radicals. The phenolic content correlated closely with the antioxidant capacity, corroborating that this phenolic class is responsible for the beneficial health effects of *Chlorella sp.* consumption. RSM proved to be effective in optimizing the extraction conditions of bioactive phenolic compounds from *Chlorella sp.* This study could be useful in the development of industrial extraction processes, including further study into the optimal number of sequential steps, to improve the efficacy of large-scale extraction systems.

## Figures and Tables

**Table 1:** Independent variables used for response surface methodology.

Standard Order	Run Order	Temperature, X_1_ (°C)	Time, X_2_ (min)	Microalgae Concentration, X_3_ (wt. %)
16	1	175	12.5	12.5
6	2	250	5	20
14	3	175	12.5	20
12	4	175	20	12.5
8	5	250	20	20
5	6	100	5	20
7	7	100	20	20
9	8	100	12.5	12.5
3	9	100	20	5
15	10	175	12.5	12.5
19	11	175	12.5	12.5
1	12	100	5	5
11	13	175	5	12.5
10	14	250	12.5	12.5
17	15	175	12.5	12.5
4	16	250	20	5
2	17	250	5	5
18	18	175	12.5	12.5
13	19	175	12.5	5
20	20	175	12.5	12.5

**Table 2:** Experimental data for the response of phenolic content and antioxidant activity under different extraction conditions.

Run Order	Phenolic Content, Y1 (mg Gallic Acid Equivalent (GAE)/g)	Antioxidant Activity, Y2 (% of Inhibition)
1	51.1	66.22
2	30.98	49.91
3	50	61.69
4	42.91	57.58
5	23.45	47.03
6	48.7	58.02
7	24.21	48.95
8	35.11	53.4
9	22.99	44.73
10	59.15	67.12
11	51.98	66.63
12	32.45	52.7
13	52.1	67.04
14	28.97	47.38
15	52.3	67.09
16	29.75	47.94
17	20.95	44.67
18	53.1	67.11
19	49.87	58.94
20	51.64	66.41

**Table 3:** Analysis of variance for response surface quadratic model (unreduced model).

Source	df	Phenolic Content			Comment	Antioxidant Activity			Comment
		Sum of Squares	F Value	Pr > F		Sum of Squares	F Value	Pr > F	
Model	9	3005.13	46.18	<0.0001	significant	1364.71	25.67	<0.0001	significant
X_I_	1	86.2	11.92	0.0062	significant	43.56	7.37	0.0217	significant
X_2_	1	175.31	24.25	0.0006	significant	68.17	11.54	0.0068	significant
X_3_	1	45.5	6.29	0.031	significant	27.62	4.68	0.0559	
X_1_X_2_	1	155.06	21.44	0.0009	significant	37.98	6.43	0.0296	significant
X_1_X_3_	1	23.6	3.26	0.101		3.39	0.57	0.466	
X_2_X_3_	1	122.93	17	0.0021	significant	6.57	1.11	0.3164	
X_1_^2^	1	986.82	136.48	0.0001	significant	452.39	76.59	0.0001	significant
X_2_^2^	1	33.27	4.6	0.0575		2.26	0.38	0.5503	
X_3_^2^	1	3.02	0.42	0.5326		23.14	3.92	0.076	
Residual	10	72.31				59.07			
Pure Error	5	44.55				0.79			
R^2^		0.9765				0.9585			

**Table 4:** Analysis of variance for the response surface quadratic model (reduced model).

Source	Sum of Squares	Phenolic Content			Comment	Sum of Squares	Antioxidant Activity			Comment
		df	F Value	Pr > F			df	F Value	Pr > F	
Model	2978.51	7	51.61	<0.0001	significant	1352.49	6	41.11	<0.0001	significant
X_I_	86.2	1	10.46	0.0072	significant	43.56	1	7.94	0.0145	significant
X_2_	175.31	1	21.27	0.0006	significant	68.17	1	12.43	0.0037	significant
X_3_	45.5	1	5.52	0.0368	significant	27.62	1	5.04	0.0429	significant
X_1_X_2_	155.06	1	18.81	0.001	significant	37.98	1	6.93	0.0207	significant
X_1_X_3_	-	-	-	-	-	-	-	-	-	-
X_2_X_3_	122.93	1	14.91	0.0023	significant	-	-	-	-	-
X_1_^2^	1196.45	1	145.13	0.0001	significant	554.67	1	101.05	0.0001	significant
X_2_^2^	47.96	1	5.82	0.0328	significant	-	-	-	-	-
X_3_^2^	-	-	-	-	-	33.61	1	6.13	0.0278	significant
Residual	98.93	12				71.29	13			
Pure Error	44.55	5				0.79	5			
R^2^	0.9679					0.9499				

**Table 5:** Mathematical equations that describe the response variables [phenolic content and antioxidant activity] in response to the extraction temperature (X_1_), time (X_2_), and solid loading (X_3_).

Response Variables	Regression Equation
Phenolic Content (mg GAE/g)	Y_1_ = 52.19 − 2.94 X_1_ − 4.19 X_2_ + 2.13 X_3_ + 4.40 X_1_X_2_ − 3.92X_2_X_3_ − 19.34 X_1_² − 3.87 X_2_²
Antioxidant Activity (% of inhibition)	Y_2_ = 65.23 − 2.09 X_1_ − 2.61 X_2_ + 1.66 X_1_X_2_ − 13.17 X_1_² − 3.24X_3_²

**Table 6:** Predicted and experimental values of responses at optimum conditions.

Response Variables	Predicted Value	Actual Value	Differences (%)
Phenolic content (mg GAE/g)	58.99	58.73	0.44
Antioxidant activity (% of inhibition)	67.17	68.05	1.31

**Table 7:** Phenolic acid constituents in some plants and algae.

Material	Extraction Solvent	Phenolic Compounds	Reference
*Spirulina platensis* (algae)	methanol/water	caffeic acid, vanillic acid, syringic acid	[31]
*Stypocaulon scoparium* (algae)	methanol, ethanol, water	caffeic acid, ferulic acid, *p*-coumaric acid, vanillic acid, gallic acid	[32]
*Spongiochloris spongiosa* (algae)	acetone, methanol	caffeic acid, *p*-coumaric acid, vanillic acid	[33]
*Amomum chinense* C. leaf	methanol	caffeic acid, ferulic acid, chlorogenic acid	[34]
Apple pomace	acetone	caffeic acid, chlorogenic acid	[35]
Litchi pulp	acetone	caffeic acid, ferulic acid, vanillic acid, syringic acid	[36]
Propolis	ethanol/water	caffeic acid, ferulic acid, *p*-coumaric acid	[37]
*Crataegi folium* leaves	acetone	caffeic acid, ferulic acid, *p*-coumaric acid, chlorogenic acid, gallic acid	[38]
Potato peel	water	caffeic acid, ferulic acid, *p*-coumaric acid, chlorogenic acid, syringic acid, gallic acid	[10]
*Chlorella sp.* (algae)	water	caffeic acid, ferulic acid, *p*-coumaric acid	This study

**Table 8:** Range of independent variables and their corresponding levels.

Symbol	Variables	Actual Value			Units
		−1	0	1	
X_1_	Temperature	100	175	250	°C
X_2_	Time	5	12.5	20	min
X_3_	Microalgae concentration	5	12.5	20	wt. %

## References

[1] Joana G.G., Villa J.A., Fernando A.J., Basilio H.J., Sepulveda D., Yahia E.M., González-Aguilar G.A. (2013). Technologies for extraction and production of bioactive compounds to be used as nutraceuticals and food ingredients: An Overview. Compr. Rev. Food Sci. Food Saf..

[2] Takaichi S. (2011). Carotenoids in Algae: Distributions, biosyntheses and functions. Mar. Drugs.

[3] Hajimahmoodi M., Faramarzi M.A., Mohammadi N., Soltani N., Oveisi M.R., Nafissi-Varcheh N. (2010). Evaluation of antioxidant properties and total phenolic contents of some strains of microalgae. J. Appl. Phycol..

[4] Herrero M., Cifuentes A., Ibanez E. (2006). Sub-and supercritical fluid extraction of functional ingredients from different natural sources: Plants, food-by-products, algae and microalgae: A review. Food Chem..

[5] Balasundram N., Sundram K., Samman S. (2006). Phenolic compounds in plants and agri-industrial by-products: Antioxidant activity, occurrence, and potential uses. Food Chem..

[6] Umar L.S., Xia W. (2005). Food phenolics, pros and cons: A review. Food Rev. Int..

[7] Fernández-Ponce M.T., Casas L., Mantell C., Rodríguez M., Martínez D.E. (2012). Extraction of antioxidant compounds from different varieties of *Mangifera indica* leaves using green technologies. J. Supercrit. Fluids.

[8] He L., Zhang X., Xu H., Xu C., Yuan F., Knez Ž., Novak Z., Gao Y. (2012). Subcritical water extraction of phenolic compounds from pomegranate (*Punica granatum L.*) seed residues and investigation into their antioxidant activities with HPLC-ABTS+ assay. Food Bioprod. Process..

[9] Wataniyakul P., Pavasant P., Goto M., Shotipruk A. (2012). Microwave pretreatment of defatted rice bran for enhanced recovery of total phenolic compounds extracted by subcritical water. Bioresour. Technol..

[10] Singh P.P., Saldaña M.D.A. (2011). Subcritical water extraction of phenolic compounds from potato peel. Food Res. Int..

[11] Khuwijitjaru P., Sayputikasikorn N., Samuhasaneetoo S., Penroj P., Siriwongwilaichat P., Adachi S. (2012). Subcritical water extraction of flavoring and phenolic compounds from cinnamon bark (*Cinnamomum zeylanicum*). J. Oleo Sci..

[12] Kim J.W., Nagaoka T., Ishida Y., Hasegawa T., Kitagawa K., Lee S.C. (2009). Subcritical water extraction of nutraceutical compounds from citrus pomaces. Sep. Sci. Technol..

[13] Jo E.K., Heo D.J., Kim J.H., Lee Y.H., Ju Y.C., Lee S.C. (2013). The effects of subcritical water treatment on antioxidant activity of golden oyster mushroom. Food Bioprocess Technol..

[14] Rodríguez-Meizoso I., Marin F.R., Herrero M., Señorans F.J., Reglero G., Cifuentes A., Ibáñez E. (2006). Subcritical water extraction of nutraceuticals with antioxidant activity from oregano. Chemical and functional characterization. J. Pharm. Biomed. Anal..

[15] Xu H., Wang W., Jiang J., Yuan F., Gao Y. (2014). Subcritical water extraction and antioxidant activity evaluation with on-line HPLC-ABTS·+ assay of phenolic compounds from marigold (*Tagetes erecta L.*) flower residues. J. Food Sci. Technol..

[16] Rodríguez-Meizoso I., Jaime L., Santoyo S., Señoráns F., Cifuentes A., Ibáñez E. (2010). Subcritical water extraction and characterization of bioactive compounds from *Haematococcus pluvialis* microalga. J. Pharm. Biomed. Anal..

[17] Lee W.C., Yusof S., Hamid N.S.A., Baharin B.S. (2006). Optimizing conditions for enzymatic clarification of banana juice using response surface methodology (RSM). J. Food Eng..

[18] Spigno G., De Faveri D.M. (2007). Antioxidants from grape stalks and marc: Influence of extraction procedure on yield, purity and antioxidant power of the extracts. J. Food Eng..

[19] Silva E.M., Rogez H., Larondelle Y. (2007). Optimization of extraction of phenolics from *Inga edulis* leaves using response surface methodology. Sep. Purif. Technol..

[20] Al-Farsi M.A., Lee C.Y. (2008). Optimization of phenolics and dietary fibre extraction from date seeds. Food Chem..

[21] Rice-Evans C., Miller N., Paganga G. (1997). Antioxidant properties of phenolic compounds. Trends Plant Sci..

[22] Koleva I.I., Van Beek T.A., Linssen J.P., Groot A.D., Evstatieva L.N. (2002). Screening of plant extracts for antioxidant activity: A comparative study on three testing methods. Phytochem. Anal..

[23] Wettasinghe M., Shahidi F. (1999). Evening Primrose Meal:  A source of natural antioxidants and scavenger of hydrogen peroxide and oxygen-derived free radicals. J. Agric. Food Chem..

[24] Naczk M., Shahidi F. (2004). Extraction and analysis of phenolics in food. J. Chromatogr. A.

[25] Cao W., Chen W., Sun S., Guo P., Song J., Tian C. (2007). Investigating the antioxidant mechanism of violacein by density functional theory method. J. Mol. Struct..

[26] Hartwig V.G., Brumovsky L.A., Fretes R.M., Boado L.S. (2012). A novel procedure to measure the antioxidant capacity of yerba maté extracts. Food Sci. Technol..

[27] Andersen O.M., Markham K.R. (2005). Flavonoids: Chemistry, Biochemistry and Applications.

[28] Ramos L., Kristenson E.M., Brinkman U.A.T. (2002). Current use of pressurised liquid extraction and subcritical water extraction in environmental analysis. J. Chromatogr. A.

[29] Ghoreishi S.M., Shahrestani R.G. (2009). Subcritical water extraction of mannitol from olive leaves. J. Food Eng..

[30] Huie C. (2002). A review of modern sample-preparation techniques for the extraction and analysis of medicinal plants. Anal. Bioanal. Chem..

[31] Klejdus B., Kopecký J., Benešová L., Vacek J. (2009). Solid-phase/supercritical-fluid extraction for liquid chromatography of phenolic compounds in freshwater microalgae and selected cyanobacterial species. J. Chromatogr. A.

[32] López A., Rico M., Rivero A., Suárez De Tangil M. (2011). The effects of solvents on the phenolic contents and antioxidant activity of *Stypocaulon scoparium* algae extracts. Food Chem..

[33] Onofrejová L., Vašíčková J., Klejdus B., Stratil P., Mišurcová L., Kráčmar S., Kopecký J., Vacek J. (2010). Bioactive phenols in algae: The application of pressurized-liquid and solid-phase extraction techniques. J. Pharm. Biomed. Anal..

[34] Butsat S., Siriamornpun S. (2016). Effect of solvent types and extraction times on phenolic and flavonoid contents and antioxidant activity in leaf extracts of *Amomum chinense C*. Int. Food Res. J..

[35] Suárez B., Álvarez Á.L., García Y.D., Barrio G.D., Lobo A.P., Parra F. (2010). Phenolic profiles, antioxidant activity and in vitro antiviral properties of apple pomace. Food Chem..

[36] Su D., Zhang R., Hou F., Zhang M., Guo J., Huang F., Deng Y., Wei Z. (2014). Comparison of the free and bound phenolic profiles and cellular antioxidant activities of litchi pulp extracts from different solvents. BMC Complem. Altern. Med..

[37] Sun C., Wu Z., Wang Z., Zhang H. (2015). Effect of ethanol/water solvents on phenolic profiles and antioxidant properties of Beijing propolis extracts. Evid.-Based Complement. Altern..

[38] Demiray S., Pintado M., Castro P. (2009). Evaluation of phenolic profiles and antioxidant activities of Turkish medicinal plants: *Tilia argentea, Crataegi folium* leaves and *Polygonum bistorta* roots. World Acad. Sci. Eng. Technol..

[39] Montgomery D.C. (1991). Experiments with a single factor: The analysis of variance. Des. Anal. Exp..

[40] Cliffe S., Fawer M.S., Maier G., Takata K., Ritter G. (1994). Enzyme assays for the phenolic content of natural juices. J. Agric. Food Chem..

[41] Blois M.S. (1958). Antioxidant determinations by the use of a stable free radical. Nature.

